# Combating QR-Code-Based Compromised Accounts in Mobile Social Networks

**DOI:** 10.3390/s16091522

**Published:** 2016-09-20

**Authors:** Dong Guo, Jian Cao, Xiaoqi Wang, Qiang Fu, Qiang Li

**Affiliations:** 1 College of Computer Science and Technology, Jilin University, Changchun 130012, China; guodong@jlu.edu.cn (D.G.); caojian13@mails.jlu.edu.cn (J.C.); xqwang15@mails.jlu.edu.cn (X.W.); fuqiang15@mails.jlu.edu.cn (Q.F.); 2 Key Laboratory of Symbolic Computation and Knowledge Engineering of Ministry of Education, Jilin University, Changchun 130012, China

**Keywords:** Cyber Physical Social Sensing, QR code, mobile social networks, compromised accounts, location-based features

## Abstract

Cyber Physical Social Sensing makes mobile social networks (MSNs) popular with users. However, such attacks are rampant as malicious URLs are spread covertly through quick response (QR) codes to control compromised accounts in MSNs to propagate malicious messages. Currently, there are generally two types of methods to identify compromised accounts in MSNs: one type is to analyze the potential threats on wireless access points and the potential threats on handheld devices’ operation systems so as to stop compromised accounts from spreading malicious messages; the other type is to apply the method of detecting compromised accounts in online social networks to MSNs. The above types of methods above focus neither on the problems of MSNs themselves nor on the interaction of sensors’ messages, which leads to the restrictiveness of platforms and the simplification of methods. In order to stop the spreading of compromised accounts in MSNs effectively, the attacks have to be traced to their sources first. Through sensors, users exchange information in MSNs and acquire information by scanning QR codes. Therefore, analyzing the traces of sensor-related information helps to identify the compromised accounts in MSNs. This paper analyzes the diversity of information sending modes of compromised accounts and normal accounts, analyzes the regularity of GPS (Global Positioning System)-based location information, and introduces the concepts of entropy and conditional entropy so as to construct an entropy-based model based on machine learning strategies. To achieve the goal, about 500,000 accounts of Sina Weibo and about 100 million corresponding messages are collected. Through the validation, the accuracy rate of the model is proved to be as high as 87.6%, and the false positive rate is only 3.7%. Meanwhile, the comparative experiments of the feature sets prove that sensor-based location information can be applied to detect the compromised accounts in MSNs.

## 1. Introduction

Cyber Physical Social Sensing makes social and virtual big data interact more with people’s daily lives, and the combination of sensors and social networks improves the development of mobile social networks (MSNs) on such handheld devices as mobile phones. Twitter’s Q1 earnings report in 2016 shows that its cost of advertising through Twitter app occupies 88% of its total investment; its average monthly active users (MAUs) were 310 million which increase 3% yearly, among whom 83% are mobile MAUs [1]. Therefore, advertising on mobile social networks and information sharing are popular with users.

However, the big data and the comparative privacy of mobile social networks attract more attackers, who can spread the malicious URLs hidden in the interactive information to users easily through sensors’ interaction on mobile devices. Currently, one of the most active ways of attacking is based on QR codes, which is simple but effective. Through the QR codes provided by businesses, the attacking codes embedded in them would be empowered to visit the social networks on mobile devices and would acquire and record the users’ information on social networks, which leads to the ever-present sending of phishing information through compromised accounts. Since such information has rich content and lots of location information, the users’ suspicion decreases, which results in the widespread use of such attacks [2].

The current malicious URL detection methods on mobile social networks mainly focus on phishing attacks, which primarily concern the security of wireless access points and the security of handheld devices’ operation systems. The methods of detecting compromised accounts are mainly applied to online social networks, which fall into three categories. The first category is to find out and analyze the features of compromised accounts’ behavior and then to construct classifiers through machine learning strategies to effectively detect the compromised accounts that are mixed in with normal accounts. The second category is to analyze the social graphs constructed by users and to apply and improve the algorithm of random walks to locate the compromised accounts in the graph. The last category is to find out the behavior model of normal accounts to spin off the accounts whose behavior model does not conform to the normal ones via the simulation and comparison of their behavior models.

However, the above methods are not obviously effective in dealing with the phishing attacks at the current mobile social networks, whose flaws are listed as follows: (1) the limitation of platforms. Since the above detecting methods mainly focus on wireless access points and handheld devices’ operation systems, the focus of them has little to do with the interaction of sensors’ information and mobile social networks themselves. Another flaw is (2) the fossilization of methods’ transplants. The simple transplant of the detection methods from online social networks leads to the lack of the research of attacking sources, attacking process and sending modes of mobile social networks. Because of the compromised accounts’ randomness of information sending behavior and because of the concealment of compromised accounts (which remain dormant after stealing users’ account information), the methods applied to online social networks are not able to identify compromised accounts effectively in MSNs, which will definitely lead to frequent false negatives.

Mobile social networks share information conveniently, and such sensors as camera and GPS are more frequently used in one’s daily life. In addition, the widespread phishing attacks in mobile social networks are all spread to users’ mobile devices through sensors. Therefore, it must be worthwhile to find a method to identify compromised accounts from the perspective of sensors. The sensors’ information will leave traces in MSNs. For example, the location information based on GPS service will be shared with every user in MSNs. Thus, sensors’ traces can be used to search and analyze effective distinguishing features to quickly detect the compromised accounts in MSNs.

This paper deals with the attacks that invade users’ devices and spread malicious URLs in MSNs by way of scanning fake QR codes of businesses. Based on the diversity of behavior, an entropy-based model is constructed for the behavior of normal accounts and compromised accounts. Then, the concepts of entropy and conditional entropy are introduced to the entropy-based model, which separately depict the diversity of users’ behavior-based features and regularity of users’ location-based features. The messages sent by normal accounts are colorful and rich in diversity, whose pictures and videos are always accompanied with texts. In addition, the location-based information shared by normal accounts tend to be regular due to the fixity of their living and working areas. However, the sending modes of fake businesses’ advertisements are very similar. Furthermore, in order to confuse the users, fake advertisements will generate false location information which will break the regularity of the users’ location information. Combining the above behavior-based features and location-based features, compromised accounts on the MSNs will be identified effectively.

Our method breaks through the limitation of existed studies, since we make up for the lack of considering of the reflection in MSNs themselves. The specific structure, different sensors, and behavior diversity are the main factors we consider for combating compromised accounts in MSNs. Sensors’ traces are sensitive and relatively accurate on social networks. We propose the location feature in mobile social activities based on GPS information. Location information is fundamental for sharing messages and feelings in real-time interaction. It also becomes the basic element for activity diversity in mobile social networks. Through considering the regularity of location information and diversity of user’ behavior, we introduce entropy and conditional entropy to build an entropy-model to evaluate the compromised accounts.

In order to testify the effectiveness of the entropy-based model proposed by this paper, we adopt machine learning strategies to evaluate the model through some evaluation parameters. We collect information sets of 489,451 accounts and 108,168,675 corresponding messages from Sina Weibo in China. After extracting the necessary features and several rounds of training, we have attained a classifier with an accuracy rate of approximately 87.6% and a false positive rate of about 3.7%. Through the comparative experiments, we confirm the effectiveness of entropy-based behavior-based features and conditional-entropy-based location-based features. The experiments prove that the classifier’s robustness is high and the results of the comparative experiments are obvious, which demonstrates that the entropy-based model can effectively detect the compromised accounts spread by QR codes.

In summary, we make three contributions as follows:

(1) Finding a widespread attacking mode disguised as businesses’ activities in MSN, whose attacking method is scanning mobile-device-based QR codes.
(2) Proposing a method based on the diversity of users’ behavior and the regularity of location information, that is, an entropy-based model to detect compromised accounts in MSNs, combining the information such sensors as GPS on mobile devices.
(3) The method proposed has a comparatively higher accuracy rate (87.6%) and lower false positive rate (3.7%). Furthermore, through the comparative experiment, it was discovered that compromised accounts in MSNs can be identified by the method based on the diversity of users’ behavior and the regularity of location information.

The rest of our paper is organized as follows: we first provide necessary related work in Section 2. Then, in Section 3, we present the dataset and a background overview which is related to detection methods. Design and analysis of the detection system is presented in Section 4. Next, we illustrate existing effective features and design our behavior-based features in detail in Section 5. We evaluate our detection system and the effectiveness of our behavior-based features in Section 6. Finally, we talk about the limitations of our method and future work in Section 7, and conclude the whole paper in Section 8.

## 2. Related Work

Due to the ubiquity of compromised accounts in mobile social networks, researchers have paid increasing attention to the investigation of compromised accounts in recent years.

Liu et al. present a coprehensive analysis on traceback apparoach, which break up the limitation on requirement of application in wireless social networks [3]. They provide an accurate marking probability analysis model for linear network, tree network, and planar network. All of them have got a better lifetime than existed studies. Yao et al. mainly focus on the QR-code-based attacks of phishing and malware that are spread through malicious URLs [4]. After analyzing the features of current scanners, they propose a new method of SafeQR to identify two weak attacks at mobile phones, which is based on and combines with the famous Google Safe Browsing API from Google company and Phishing Attack API. This method devises and applies visual warning schemes to warn the current users. Although this method overcomes the limitation of the previous methods that only check the net of access points, the API they adopt becomes the bottleneck in increasing its real-time performance and decreasing its false negative rate. Vidas et al. make two experiments to investigate the feasibility of spreading QR-code-initiated phishing (or QPhishing) [2]. The results of their experiments show that curiosity is always the main force that drives common people to scan unknown QR codes, 85 of whom will continue to visit the URLs in QR codes, which indicates that phishing attacks in QR codes are easily spread quickly.

Wu et al. point out that the existing methods are mainly PC-based which are not applicable to handheld mobile devices, so they propose a lightweight application that is applicable to mobile phones to defend phishing: MobiFish [5]. This application compares its actual identity and the identity of other websites and applications so as to identify the real websites and redirection. This application has been deployed in Nexus4 (An mobile designed by Google). Combining with Facebook, the application’s performance is proved to be high. Marforio et al. classify the phishing attacks on platforms of mobile phones and classify their corresponding methods [6]. Then, they point out that the existing studies neglect the investigation and analysis of security indicators of both attackers and victims, so they propose personalized security indicators to decrease the effectiveness of phishing attacks in MSNs. They analyze a great number of users’ features and compare the experiments’ results, which clearly indicate that personalized security indicators are necessary in detecting phishing attacks. Liu et al. design ActiveTrust to deal with the challenge based on active detection-based security and trust [7]. The approach could fully use the energy with the routes’ generation and distribution. According to the comprehensice throretical and results from experiments, ActiveTrust could effectively against the black hole attacks. Kim et al. mainly research MITM (Man-In-The-Middle) phishing attacks, which means attackers that hide between servers and users [8]. MITM may hide for long and substitute phishing URLs by operating authentication and trading information. These kind of attacks last for a long time, but they are not easily discovered. Kim et al. propose a geo-location-based QR code authentication mechanism to detect and stop ART (Active Real-Time) MITM phishing attacks. However, this mechanism may only be on guard against the malicious URLs, and it can not identify the attacks that send information via pictures.

Much existing research has addressed the problem of compromised account detection on online social networks. This research tries to solve the problem by two methods. One is to identify compromised accounts themselves [9,10], and the other is to identify spam messages sent by them so as to identify compromised accounts [11]. Previous research using the first method mainly makes use of the features that compromised accounts have but differ from those of normal accounts, including account properties, behaviors, and message contents. Chu et al. [10] observe the differences among human, bot, and cyborg in term of tweeting behavior, tweet content, and account properties, and propose a classification system based on machine learning strategies to determine a user’s likelihood of being a human, bot or cyborg. Egele et al. [9] present a novel approach to detect compromised accounts on social networks. They build a statistical model based on users’ behavior and messages, and use an anomaly detection system to identify accounts that experience a sudden change in behavior. Some research using the first method also exploit the network structure of spammers on online social networks, and propose some graph-based algorithms to identify spammers. Tan et al. [12] first propose a Sybil-defense-based spammer detection scheme SD2 by taking the social network relationship into consideration, and then design an unsupervised spam detection scheme called UNIK in order to make it highly robust in facing an increasing level of spam attacks. However, spammers on social networks also change their features to evade the existing detection methods. Yang et al. [13] make a comprehensive and empirical analysis of the evasion tactics utilized by Twitter spammers and then design several new detection features to detect more Twitter spammers. The existing research using the second method utilizes text-based features of spam messages. Gao et al. [14] present an online spam filtering system, and propose reconstructing spam messages into groups for classification. Dong et al. propose RMER, an interesting event data collection approach [15]. In order to increase the network lifetime and detection efficiency, they decrease the monitor nodes that close to the Sink. Meanwhile, the RMER could effectively converge mulitipath routes into a one-path route of event monitoring nodes.

Different from the previous work, we propose using behavior diversity to distinguish compromised accounts from normal ones in MSNs. Our work focuses on location regularity and behavior diversity in different types of messages. We also propose entropy and conditional entropy to present the diversity and regularity. Through the analysis of these evaluation parameters, we demonstrate that behavior diversity is highly effective in detecting compromised accounts in MSNs.

## 3. Background and Dataset

In this section, we first present a background to get a broader view of the detection methods, including the description of QR code, Sina Weibo, forwarding, topics (“#”), and replying to someone (“@”) in messages, etc. Then, we introduce our dataset, which is the basis for achieving our goal. We will discuss the dataset in two subchapters, dataset collection and identifying.

### 3.1. Background Overview

In order to analyze the behavior of accounts’ message sending, we need to present basic information related to our methods. As our method focuses on attacks based on QR codes in Sina Weibo, we introduce QR code, Sina Weibo, and some other typical functions in Sina Weibo.

***QR Code:*** quick response code. QR codes can send messages very quickly, and they are also very reliable with low cost and high capacity for messages. The information inside QR codes is various, for example, redirection link, location information, time stamp, IDs of the corresponding platform’s users, all of which can not be identified in the pictures of QR codes. Only if the users scan the QR codes by their handheld mobile devices and the QR codes are decoded can the targeted website be redirected. Because of the opacity of encoding and decoding of QR codes, users have no idea about whether the information is secure or not, which increases the possibility of the attacks at users and makes users’ sensitive information be leaked out, hence indirectly spreading such attacks as phishing and malware. The existing attacking modes of QR codes include MITM, Structured Query Language (SQL) injection, XSS (cross site scripting), command injection, etc. These attack modes are not easy to be discovered, especially when attackers act as the generator of QR codes, which makes it impossible for users to defend effectively. URLs in QR codes will redirect the users to the phishing websites where false businesses are active, which, of course, results in the economic loss of users. Furthermore, the URLs in QR codes comprise the information that can be injected into browsers, which will make it easy to attack users’ browsers and devices and will open this device’s authority to attackers, hence more leakage of information. Therefore, QR codes are not only the window through which the information flows in, but also a necessary way to spread attacks at handheld mobile devices [16].

***Sina Weibo:*** Sina Weibo is one of the largest micro-websites in China. It provides an online community that is open to the public to talk about the latest news. As it has more than 70 million daily active users, each topic or current news item may have a huge number of responses in different areas. Because of the limitation of Chinese characters in Sina Weibo, messages are short for people to read. However, there are still such security issues as compromised accounts trying to hide attacks in suspicious URLs in messages that are sent. Sina Weibo was attacked by XSS in 2011, creating a lot of confusion for people; therefore, ensuring privacy and security in Sina Weibo is necessary.

***Forward:*** There is a function named “forward” in Sina Weibo, which is almost the same as the function “retweet” in Twitter. However, the forward function in Sina Weibo contains the original message, and this is different from retweet in Twitter. If a message has been forwarded, people can easily look at the original message. Messages forwarded in one circle can be seen as power-law distribution. Although forwarding messages is considered convenient and is widespread, unfortunately, compromised accounts also focus on this function. Compromised accounts utilize it to propagate advertisements or suspicious URLs in hot messages.

***Replying:*** Replying in a message or private message can make users notice the message. This symbol (“@”) is often used among friends who are in the same interest group or among friends in real-life. This function has made it convenient for people to communicate with a target person or to share opinions. However, utilizing this reply symbol (“@”) also gives compromised accounts a great cheating power based on users’ trust. Compromised accounts often utilize this symbol to randomly select people in the current page to get them to read and click on suspicious URLs of compromised accounts’ information.

### 3.2. Dataset

To achieve our goal of detecting compromised accounts in MSNs, we need to collect real-time data before analysis. Since Sina Weibo is one of the largest micro-websites in China, which has many compromised accounts on it, we use Sina Weibo as our source of real-time data. After collection, we still need to identify compromised accounts from millions of accounts in order to have a comparison for subsequent data analysis.

***Data Collection:*** Sina Weibo provides free API [17] for researchers to collect information of accounts and messages. We build a collection crawler based on Python to collect a real-time dataset. First, we take several account seeds randomly from different cycles in order to replicate a setting close to natural distribution. Then, we use the resultant list of account seeds as new seeds to spread by crawling. Not only users’ information, but also messages that they send will be collected concurrently. Features of both accounts’ information and corresponding messages are needed to train a classifier. Preliminary data collection from online social networks is difficult to be applied now. Because of privacy and protection of personal information, it is hard for researchers to crawl for as much data as they want, especially for big data. Thus, after a long time, we collect approximately 500,000 accounts and about 100 million corresponding messages.

***Labeling:*** In order to have an effective analysis of compromised and normal accounts, we need to distinguish and label them first. We transfer URLs extracted from corresponding messages to Google safe browsing API [18] and Virustotal (Virustotal public API v2.0, Google, Dublin, d.b.a, Ireland) [19] to create official labels. If URLs from one account have been labeled by these two websites more than five times, we will consider this account as a compromised one. After this step, we still need to judge strange accounts independently. Our team selected random accounts to label. Eventually, we labeled approximately 2000 compromised accounts, and 2000 normal accounts.

## 4. System Overview

**Figure 1:**
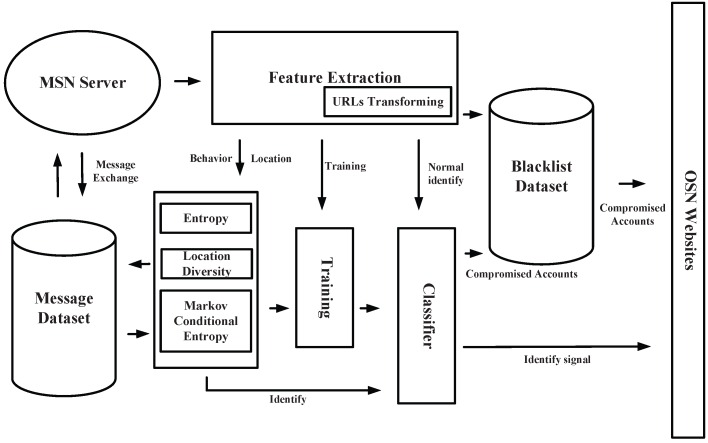
System overview.

**Figure 2:**
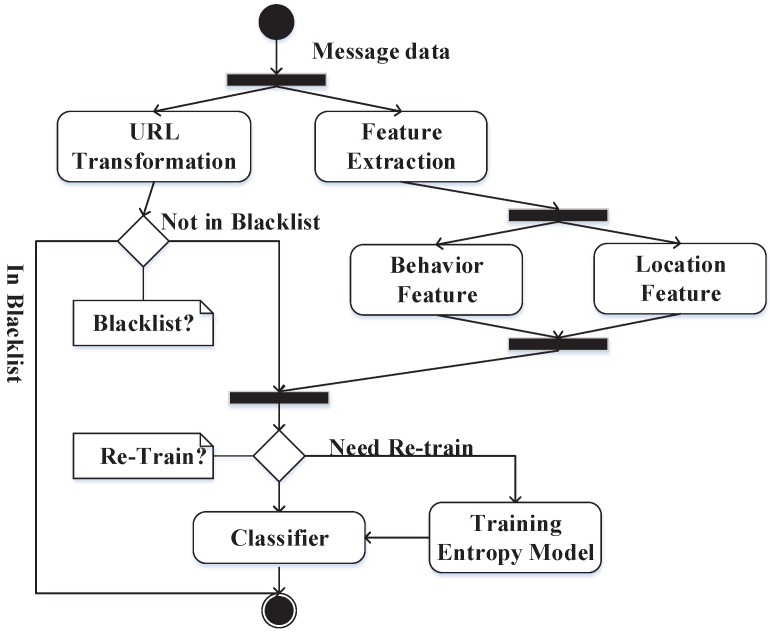
Activities of message data.

In this section, we plan to introduce every part of our detection system in detail on mobile social networks. We built a detection system based on machine learning strategies, which can be viewed in Figure 1. This system has mainly about seven parts. We have also drawn a figure of the unified modeling language (UML) activity (Figure 2) with our method that would help understand the inner flow of message data. The flow of data in the models is similar to that of other systems that are based on machine learning strategies. However, we include our own elements of the behavior-based features and plan to introduce these elements into the system one by one.

***Feature Extraction:*** This part is the first to be encountered when a message stream comes from an MSN server. In this part, we utilize multi-process and the Sina Weibo API to extract the basic features that we need to handle. As features in our solutions are not simple, we need to calculate these basic features in the next few parts. Features about behavior will be sent to entropy parts, which can calculate the value of entropy and conditional entropy based on a Markov model. Accounts and URLs in messages will first be sent to the Blacklist Dataset.

***URLs Transforming:*** This is an accessorial part of Feature Extraction. Because of the limitation of 140 Chinese characters for messages in Sina Weibo, URLs in each message have been shortened by an official server. Attempts are made to transform these short URLs back to the original ones based on Sina Weibo URL APIs.

***Blacklist Dataset:*** This part is set for saving time in detecting existing compromised accounts and suspicious URLs. We built a blacklist based on an existing blacklist from official sources. This dataset has two main indexes, one is account id, and the other is URLs in blacklist. If a URL has been identified as a suspicious one, we will put its corresponding accounts into this dataset. Then, accounts from where messages containing these URLs are sent immediately will be identified as compromised accounts. In addition, if an account is matched with the one in this dataset, this account will be identified. If none of the accounts or URLs have been matched in this part, features should be calculated and transferred to the classifier.

***Entropy Model:*** This part is based on the behavior-based features proposed by us. We propose utilizing entropy and conditional entropy to present behavioral diversity in sending messages in MSNs. We get features, which are needed to calculate the value of entropy and conditional entropy, from Feature Extraction parts. The values will be seen as behavior-based features for further training and identifying. In the process of building the Markov model, we still need to take several messages from the sending list of corresponding accounts. We would like to calculate a value of conditional entropy for future identification based on machine learning.

***Location Diversity:*** This part is about an identification model based on location diversity of such mobile devices as mobile phones. By introducing the location-based features, location information is further processed in this model to identify whether this location information can form a normal information loop in a certain period of time or to identify whether the location information has been manipulated, or to identify the false location information itself which does not appear frequently in this account. Through the processing of this model, the location-based feature sets can be trained comprehensively with the behavior-based feature sets, so as to generate an effective classifier for hidden compromised accounts. The introduction of this model can be combined with the entropy model part, which can stop malicious URLs (spread through businesses’ activities) from their sources so as to protect users’ privacy and ensure their information’s security.

***Message Dataset:*** This is not a high-priority part for part for building Markov models in order to calculate the value of conditional entropy. Account datasets first include all vectors from the Feature Extraction model. Meanwhile, this kind of datasets can be used as a cache dataset for getting existing information of accounts from the Sina Weibo server through API. As multiple accounts need to be identified at the same time, we need to use a cache vector to collect corresponding information of the target accounts. Meanwhile, information about this account will be cleaned in this dataset for privacy.

***Training:*** This part periodically runs because the effectiveness of the system in detecting compromised accounts may weaken with time. Features’ vectors will be sent to this part for training an effective classifier through machine learning strategies. In this part, we utilize several effective machine learning strategies, such as Random Forest, Support Vector Machine (SVM), etc.

***Classifier:*** This part is for identifying compromised accounts in a normal message stream. The Classifier built in the Training part over a period of time will be updated in this part. Features for identifying them will be sent to this part together. If an account has been identified as compromised, the message will be labeled with a signal of insecurity and the account will be sent to Blacklist Dataset for subsequent identification.

Overall, our system needs to be deployed on the server to identify and stop QR-code-based compromised accounts on mobile social networks. The Behavior-Based Feature Model part is the key part for detecting compromised accounts, and we would like to see it does form an exhaustive line of defense for privacy and security on mobile social networks.

## 5. Features Illustration

In this section, we illustrate the behavior-based features that we propose above. Then, we plan to introduce several effective features that have been used in previous studies. These existing features will be combined to build an effective classifier, together with the entropy-based features we propose.

### 5.1. Behavior-Based Feature

In this part, we are going to illustrate the behavior-based features we propose. We have said to utilize entropy and conditional entropy to present behavior diversity and location regularity when sending messages on mobile social networks. Normal accounts and compromised accounts have different entropy-based models of location distribution when sending messages. The entropy-based models of normal accounts are much more diverse, while compromised accounts have a simple model in sending messages. First, for the purpose of preliminary calculation, we plan to introduce entropy and some basic items when sending messages. Then, we need to build a Markov model to calculate conditional entropy. The values of entropy and conditional entropy are the behavior-based features that we propose. Finally, we would like to propose the location distribution of entropy-based models.

**Figure 3:**
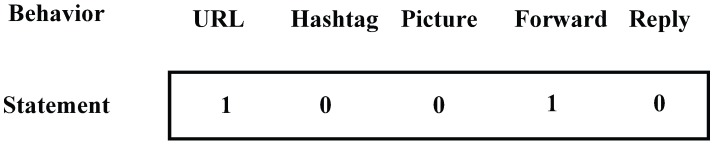
Sample of items in sending messages.

Entropy: This item is the typical one to measure the confusion degree of statistics. We have introduced it to present the behavior diversity when sending messages on mobile social networks. The bigger this value, the more diverse the behavior. In order to calculate this value, we need to make it clear which category the message falls into. We choose five items: URL, Picture, Hashtag, Forward, and Reply. We take a five-bit integer to represent the category of each message, which can be seen in Figure 3, and every bit matches with the corresponding item. If the message sent contains any behavioral item, the corresponding bit of it will be set to 1, otherwise, it will remain at 0 at the initial stage. Then, the category of all messages sent can be seen in this integer. We calculate the entropy of a user’s behavior based on Formula (1), in which $p_{i}$ represents the probability of messages belonging to category *i*:

(1) $$H\left(X\right)=-\sum_{i=1}^{n}p_{i}\log_{2}p_{i}.$$

**Figure 4:**
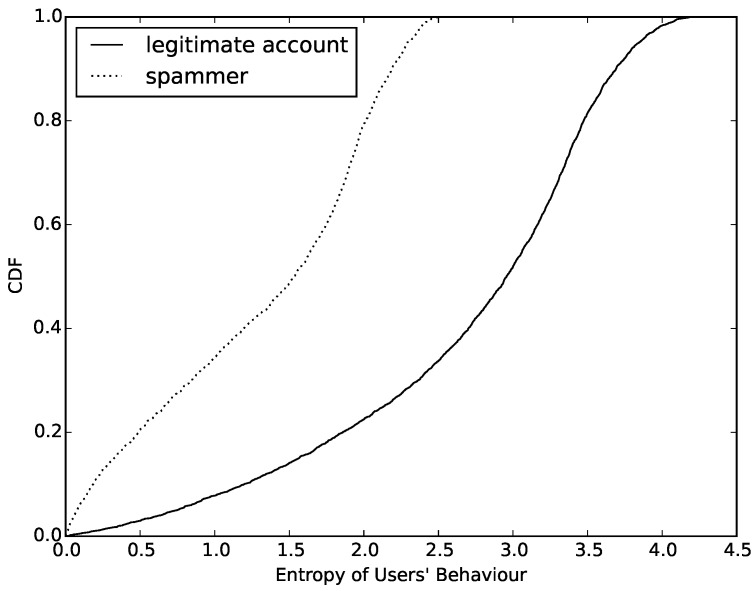
Entropy of users’ behavior.

According to Formula (1), we calculate the value of entropy of normal accounts and compromised accounts. Then, we count the number of different values and draw a cumulative distribution function (CDF), which can be seen in Figure 4. Observing this figure, we determine that the value of entropy based on behavior can easily distinguish compromised ones from normal accounts. First, more than 65% of normal accounts have a value higher than 2.5. However, the entropy value of compromised ones are almost always lower than 2.5. This is an obvious threshold in entropy between compromised and normal accounts. Furthermore, about 22% of normal accounts have an entropy value lower than 2.0. We try to find out the reasons for this phenomenon and find that most of them are sent by cyborg and most of them have the same behavior model when sending messages. Finally, we also find that more than half of the compromised accounts have an entropy value between 1.5 and 2.5. After analyzing the samples whose entropy values are in this interval, we find that most of these compromised accounts are trying to change their behavior model periodically in order to evade detection.

In summary, we still need to consider behavior-based features more carefully, considering the evasion tactics of compromised accounts. Then, we introduce conditional entropy to prevent periodically changing behavior models of compromised accounts.

We introduce conditional entropy to deal with the shortfalls of entropy because of periodical changes in sending behaviors of compromised ones. Conditional entropy is a typical measure in information theory. If the value of conditional entropy is higher, it means that the behavior model of message sending is simpler. Otherwise, it means the behavior model of message sending is more diverse. In order to get this value, we use Markov models for calculation. At first, we need to introduce Formula (2), which is utilized for calculation:

(2) H(Y|X)=H(X,Y)-H(X).

**Figure 5:**
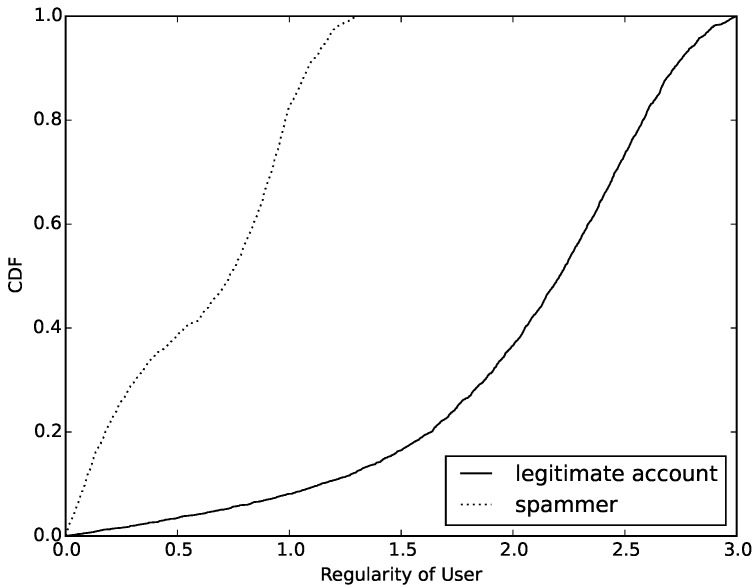
Regularity of users’ behavior.

According to Formula (2), we need to determine the joint entropy H(X,Y) in order to calculate conditional entropy. We introduce transition probabilities in the Markov model to deal with this problem. After transferring our dataset samples into the Markov model, we determine the final value of conditional entropy, and then we draw a figure based on the values, which can be seen in Figure 5.

After observing this figure, we can see that the values of conditional entropy in most compromised accounts are lower than 1.0. However, for normal accounts, no more than 10% has a value lower than 1.0. Of course, this result is based on the same entropy value for compromised accounts and normal accounts. This result of comparison clearly proves that compromised accounts have a much simpler model of message sending than normal accounts. Through this conditional entropy, we can identify disguised compromised accounts that are good at changing their behavior model in message sending in mobile social networks.

Through the comprehensive consideration of the entropy model of behavior diversity, we show our algorithm, which is used to calculate the entropy of feature sequence with pseudo codes (Algorithm 1). Observing from Figure 4 and Figure 5, both of these features are good at distinguishing behavior models of message sending on mobile social networks. We will put them together with existing effective features to build a classifier to detect compromised accounts.

**Algorithm 1:** Calculating the entropy of feature sequence. **Require:**  $S_{n}$: Feature sequence;  *n*: Length of the sequence;  *m*: Maximum of all elements in $S_{n}$. **Ensure:**  $H(S_{n})$: Entropy of the sequence.
1:Initialize each element of array D[0...m] as 0;2:**for** each i∈[1,n] **do**3: $D\left[S_{i}\right]=D\left[S_{i}\right]+1$;4:**end for**5:$H(S_{n})=0$;6:**for** each i∈[0,m] **do**7: **if** D[i]≠0 **then**8:  $H\left(S_{n}\right)=H\left(S_{n}\right)-\frac{D[i]}{n}\log_{2}\frac{D[i]}{n}$;9: **end if**10:**end for**11:**return** $H(S_{n})$;

### 5.2. Location-Based Feature

We have introduced the ways to classify users’ behavior. Likewise, if we want to analyze the diversity of location-based features, we have to classify users’ location-based features first. Since normal users’ locations tend to be frequently adjacent to each other, the method we use to classify location-based features must be able to put the features of the adjacent locations into the same category. Therefore, we use the clustering algorithm of *K*-means, which select K points (the total number of the points is K) randomly as clustering centers, and select the cluster each point belongs to in every iteration for the purpose of making the value of *J* become the smallest:

(3) $$J=\sum_{j=1}^{n}\sum_{k=1}^{K}u_{kj}\left|\right|x_{j}-z_{k}{\left|\right|}^{2},$$

where $u_{kj}$ indicates whether point $x_{j}$ belongs to cluster *k*, and $z_{k}$ represents the center of the $k_{th}$ cluster. In other words, every point is to be put into the cluster whose center is the nearest to it, and then the center of this cluster will be updated to the average value of all the points in this cluster. When the center does not change any more or the total distance between the center and all the points has no obvious change, the algorithm stops working.

Then, we need to select a validity index to evaluate the clustering result when different value of k is selected. We use the Calinski–Harabase (CH) index. For *n* points and dataset of K clusters, CH index is:

(4) $$\frac{trace\;B/(K-1)}{trace\;W/(n-K)}.$$

In this formula, *B* is the scatter matrices between clusters, and *W* is the scatter matrices within clusters. The higher the CH index value, the better result the clustering and the closer to the reality. The trace of scatter matrices between clusters can be expressed as:

(5) $$trace\;B=\sum_{k=1}^{K}n_{k}\left|\right|z_{k}-z{\left|\right|}^{2},$$

where $n_{k}$ represents the point number of the $k_{th}$ cluster, $z_{k}$ represents the central point of the $k_{th}$ cluster, and *z* represents the center of all points. Scatter matrices within clusters can be expressed as:

(6) $$trace\;W=\sum_{k=1}^{K}\sum_{i=1}^{n_{k}}\left|\right|x_{i}-z_{k}{\left|\right|}^{2}.$$

Hence, the value of CH index can be expressed as:

(7) $$CH=\left[\frac{\sum_{k=1}^{K}n_{k}\left|\right|z_{k}-z{\left|\right|}^{2}}{K-1}\right]/\left[\frac{\sum_{k=1}^{K}\sum_{i=1}^{n_{k}}\left|\right|x_{i}-z_{k}{\left|\right|}^{2}}{n-K}\right].$$

From the above formulas, we can see that the denominator represents the scatter matrices between clusters; hence, the larger the denominator, the more obvious the difference between all clusters. The numerator represents the scatter matrices of the points within every cluster; hence, the smaller the numerator, the more similar the points in the same cluster. Therefore, the larger the CH index value, the better the whole clustering. We cluster every user’s location-based features by the above formulas and use the CH index to evaluate the result of clustering. Then, the highest value of CH index is chosen out and its number of clusters is set at K, which are labeled as 0, … , K−1; thus, every location-based feature of a certain user is classified, and the label of the cluster it belongs to is the category its feature falls into. Since we use the clustering algorithm, the features of the adjacent locations will be put into the same category, which is beneficial for our analysis and detection.

**Figure 6:**
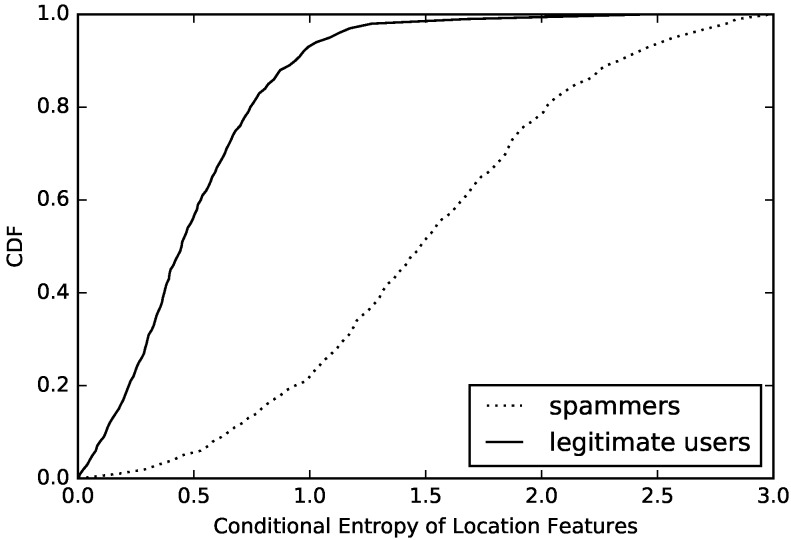
Conditional entropy of location-based features.

**Figure 7:**
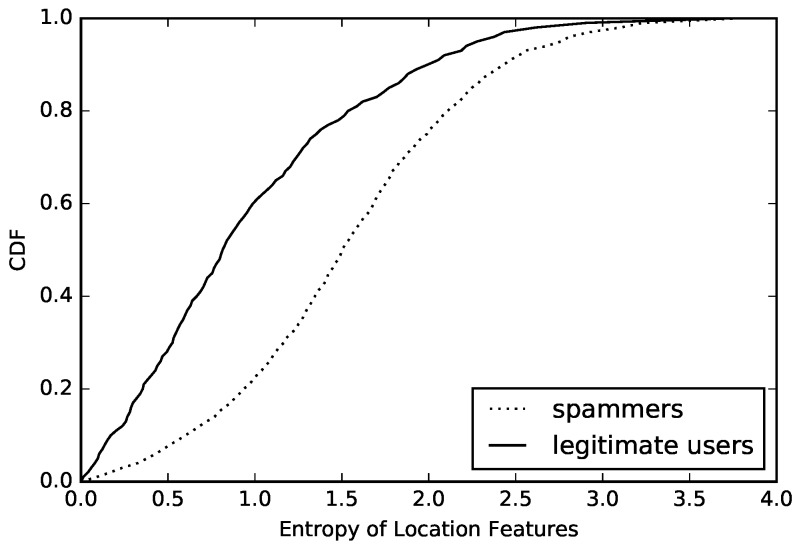
Entropy of location-based features.

We also analyze the diversity and regularity of users’ location-based features in sample’s data, calculate the entropy and conditional entropy of every user’s location-based features, and then draw the CDF of compromised accounts and normal accounts, which are presented in Figure 6 and Figure 7. The above figures show that the entropy and conditional entropy of compromised accounts’ location-based features are lower than those of normal accounts’ location-based features, which are different from behavior-based features but agrees with our intuition: normal users often appear in fixed positions, so their location features are of low diversity and high regularity, while location-based features of compromised accounts are randomly generated, hence the high diversity and low regularity which is opposite to that of normal accounts. However, what is similar to that of behavior-based features is that the difference of location-based features’ regularity between compromised accounts and normal accounts is more obvious than the difference of location-based features’ diversity between compromised accounts and normal accounts. As we see, location-based features’ entropy of 60% compromised accounts is below 1.8, while location-based features’ conditional entropy of 53% compromised accounts is below 1.8. The location-based features’ entropy of 40% normal accounts is above 1.0, while the location-based features’ conditional entropy of 9% normal accounts is above 1.0.

We show our algorithm, which is used to calculate the conditional entropy with pseudo codes (Algorithm 2) based on the methods mentioned before. Through the analysis of diversity and regularity of location-based features, we can draw a similar conclusion to that of behavior-based features: there are obvious differences in the diversity and regularity between compromised accounts and normal accounts, which can be used to detect compromised accounts.

**Algorithm 2:** Calculating the Conditional Entropy of Feature Sequence. **Require:**  $S_{n}$: Feature sequence;  *n*: Length of the sequence;  *m*: Maximum of all elements in $S_{n}$. **Ensure:**  $H(S_{n+1}|S_{n})$: Conditional entropy of the sequence.
1:Initialize each element of array T[0..m,0...m] as 0;2:**for** each i∈[1,n-1] **do**3: $T\left[S_{i},S_{i+1}\right]=T\left[S_{i},S_{i+1}\right]+1$;4:**end for**5:$H(S_{n},S_{n+1})=0$;6:Calculate $H(S_{n})$ using Algorithm 1;7:**for** each i∈[0,m] **do**8: **for** each j∈[0,m] **do**9:  **if** T[i,j]≠0 **then**10:   $H\left(S_{n},S_{n+1}\right)=H\left(S_{n},S_{n+1}\right)-\frac{T[i,j]}{n-1}\log_{2}\frac{T[i,j]}{n-1}$;11:  **end if**12: **end for**13:**end for**14:**return** $H\left(S_{n+1}|S_{n}\right)=H\left(S_{n},S_{n+1}\right)-H\left(S_{n}\right)$

### 5.3. Existing Features

We also make use of seven other features, which are based on accounts and messages. These features have been proved to be effective in previous studies and will be combined with our method (which uses behavior-based features) to train a classifier.

We can see those existing features in Table 1, along with behavior-based features. Although there are several other features, we integrate only these effective features in our work. Then, we transfer the value of these features from a labeled dataset to train a classifier.

## 6. Evaluation

In this section, we plan to prove the effectiveness of our detection system in mobile social networks, including the effectiveness of the above model of behavior-based features. Finally, we compare the effectiveness of the systems with and systems without the proposed behavior-based feature model.

### 6.1. Evaluation of Detection System

In this section, we plan to evaluate our detection system based on the data from Sina Weibo, which we have collected and labeled. First, we utilize several machine learning strategies to train an effective classifier, and then we put our testing samples into the classifier for evaluation.

After labeling, we have about 2000 normal accounts and 2000 compromised accounts. Next, we conduct ten-fold cross-validation in Weka 3.7 on different machine learning strategies, such as J48, Random Forest, and Support Vector Machine (SVM). Accuracy rate and false positive rate can be seen in Table 2, which is based on Table 1, and we will take an average of these to be the final value. Ultimately, the accuracy rate of the detection system is about 87.6%, and the false positive rate is about 3.7%.

According to the labeled samples, we analyze the reason for the above results. After observing the false positive labels and false negative labels, we find that the labeled samples can not be assured of absolute labeling accuracy. Labels based on the blacklist need to be re-labeled to ensure a higher accuracy rate and lower false positive rate.

### 6.2. Evaluation of Entropy-Based Model’s Effectiveness

In this section, we plan to evaluate the effectiveness of the entropy-based model proposed by us. First, we classify the values of features that have been extracted previously. Then, we use features’ sets with and without entropy-based features to re-train and evaluate. We still use ten-fold cross-validation based on machine learning stategies, such as J48, Random Forest, and SVM. The accuracy rates and false positive rates for effectiveness of behavior-based features can be seen in Table 3.

According to the results in Table 3, the behavior-based feature set is much more effective in detecting compromised accounts in MSNs. As we have proposed above, behavior-based features are very important for presenting behavior diversity when sending messages. Although there are some ups and downs in these machine learning stategies, based on these behavior-based features, detection systems can achieve a higher accuracy rate and a lower false positive rate. This is enough for proving the effectiveness of the proposed behavior-based features model.

The values of accuracy rate and false positive rate for effectiveness of location-based model can be seen in Table 4. From the analysis of the comparative experiments’ results in Table 4, location-based features can effectively assist entropy-based model to detect compromised accounts in MSNs. Meanwhile, from the analysis of false negative rate, the introduction of the concept of sensor-based location information’s regularity can make it effective to distinguish compromised accounts from normal accounts. Most behavior of compromised accounts is not different from that of normal accounts; therefore, the behavior-based feature set of normal accounts can not be used to filter compromised accounts. Only through identifying sensor-based features can we discover the false information and its location information so as to identify compromised accounts in MSNs.

## 7. Limitation and Future Work

This section discusses limitations of our detection method, and then presents the future work planned to perfect our detection method.

Comparison with the previous research is challenging. We can not reconstruct all the existing methods in detail because of the differences in datasets and source codes. We need to take a completely different approach to prove the effectiveness of proposed behavior-based features. Finally, additional features of behavior still need to be added because the features we propose are limited. There are several other features, which can be used to increase the accuracy rate of our detection method.

For future work, we first need to collect much more data from different MSNs. We will communicate with official platforms and cooperate with them to acquire more data. Meanwhile, we will install our detection method on different MSNs to make sure our method is applicable to different platforms. Then, we will communicate with authors of prior research to get their samples and source codes for comparison. It is essential to compare with existing studies in order to perfect our detection method. In addition, we will perfect our detection method by continuous self-learning, such as feedback training. Finally, we will try to re-analyze the dataset to identify other features that are related to behavior-based features. We hope we can perfect our method and present a more effective detection method for the application on mobile social networks.

## 8. Conclusions

Cyber Physical Social Sensing makes MSNs (based on the sharing and interaction of sensors’ information) become popular with a great number of users. However, we can not ignore the attacks that send disguised malicious URLs by scanning QR codes to control compromised accounts. This paper tries to find out the source of compromised accounts, to analyze the location traces based on sensor information, and introduce the concepts of entropy and conditional entropy so as to construct an entropy-based model to effectively identify compromised accounts in MSNs. The performance of this model is proved to be high by cross-validation. Through the comparative experiments of location-based features and through analyzing the experiments’ data of false positive rates and false negative rates, we find that location-based features are better for locating and detecting compromised accounts in MSNs. The method proposed by this paper makes up for the limitations of existing methods: platforms’ restrictiveness and solutions’ simplification; thus, a better cyberspace of mobile social information sharing is constructed for users.

## Figures and Tables

**Table 1:** Feature sets.

Without Behavior Feature	Number of fans	Friends in following list of accounts
	Account reputation	Ratio between number of fans and sum of related friends
	Age	Age of accounts
	URL ratio	Ratio between number of URLs in sending messages of latest one week by accounts and sum of number of messages
	Hashtag (#) ratio	Ratio between number of hashtags (#) in sent messages of latest one week by accounts and sum of number of messages
	Reply (@) ratio	Ratio between number of reply (@) characters in sent messages of latest one week by accounts and sum of number of messages
	Forward ratio	Ratio between number of forward in sending messages of latest one week by accounts to sum of number of messages

**Table 2:** Performance of entropy-based classifier.

Classifiers	Accuracy Rate	False Positive Rate
J48	89.6%	4.9%
Random Forest	93.7%	2.1%
SVM	91.2%	3.3%

**Table 3:** Evaluation on effective of behavior-based features.

	Accuracy Rate	False Positive Rate
Without Behavior-Based Feature	85.4%	7.2%
With Behavior-Based Feature	91.5%	3.4%

**Table 4:** Evaluation of location-based features’ effectiveness.

	Accuracy Rate	False Positive Rate
Without Location-Based Feature	82.7%	8.9%
With Location-Based Feature	87.6%	3.6%
